# Genotypes and phenotypes of neurofibromatosis type 1 patients in Japan: A Hereditary Tumor Cohort Study

**DOI:** 10.1038/s41439-024-00299-4

**Published:** 2024-11-26

**Authors:** Mashu Futagawa, Tetsuya Okazaki, Eiji Nakata, Chika Fukano, Risa Osumi, Fumino Kato, Yusaku Urakawa, Hideki Yamamoto, Toshifumi Ozaki, Akira Hirasawa

**Affiliations:** 1 Department of Clinical Genomic Medicine, Okayama University Graduate School of Medicine, Dentistry and Pharmaceutical Sciences, Okayama, Japan; 2 Department of Orthopedic Surgery, Okayama University Hospital, Okayama, Japan; 3 Department of Clinical Genetics and Genomic Medicine, Okayama University Hospital, Okayama, Japan; 4 Department of Genetic Medicine, School of Medicine, Fujita Health University, Aichi, Japan

**Keywords:** Genetic testing, Cancer genetics, Disease genetics

## Abstract

Neurofibromatosis type 1 (NF1) presents with a broad spectrum of clinical manifestations, including an increased risk of tumor development and hypertension. Comprehensive data on genotype‒phenotype correlations in patients with NF1 are limited. Therefore, in this study, we aimed to elucidate the detailed genetic and clinical characteristics of NF1 in a hereditary tumor cohort. We performed sequencing and copy number assays in a clinical laboratory and analyzed the clinical data of 44 patients with suspected NF1. Germline pathogenic variants were detected in 36 patients (81.8%), and 20.7% of the variants were novel. Notably, 40.0% of adult patients presented with malignancies; female breast cancer occurred in 20.0% of patients, which was a higher rate than that previously reported. Hypertension was observed in 30.6% of the adult patients, with one patient experiencing sudden death and another developing pheochromocytoma. Three patients with large deletions in *NF1* exhibited prominent cutaneous, skeletal, and neurological manifestations. These results highlight the importance of regular surveillance, particularly for patients with malignancies and hypertension. Our findings provide valuable insights for genetic counseling and clinical management, highlighting the multiple health risks associated with NF1 and the need for comprehensive and multidisciplinary care.

## NF1 Patients Face Increased Cancer and Hypertension Risks

Neurofibromatosis type 1 (NF1) is a genetic disorder that causes skin changes and tumors. This study explores the genetic and physical characteristics of NF1 in Japanese patients. Researchers collected data from 44 patients with suspected NF1 between 2020 and 2024. They used genetic tests to identify changes in the *NF1* gene, which is crucial for tumor suppression. The study found that 92.3% of suspected patients with NF1 had detectable genetic changes linked to NF1. These changes included various types of variants, such as truncating, missence and large deletion variants. The study showed that patients with large deletion had more severe symptoms, including tumors and developmental issues. The findings suggest that understanding these genetic differences can help improve patient care and monitoring. In conclusion, this research highlights the importance of genetic testing in managing NF1. This summary was initially drafted using artificial intelligence, then revised and fact-checked by the author.

## Introduction

Neurofibromatosis type 1 (NF1) is characterized by diverse clinical features, including multiple café-au-lait spots, skinfold freckling, iris Lisch nodules, and tumors of the nervous system^1^. Historically, NF1 has been diagnosed according to clinical criteria proposed by the National Institutes of Health (NIH) in 1988^2^. In Japan, revised NIH criteria were utilized starting in 2018^3^. However, *NF1* genetic testing has recently become clinically available in many countries because of its high detection rates and low cost. The *NF1* gene, located on chromosome 17q11.2, plays a crucial role in tumor suppression, and pathogenic variants in this gene lead to the development of NF1^4^. Recent studies have shown that specific variant types are associated with a greater prevalence of severe phenotypes in patients with NF1^5–8^. For example, *NF1* microdeletion syndrome and missense variants in codons 844–848 have been linked to more severe phenotypes, as well as a greater tumor burden and increased risk of malignancy^9,10^. These findings highlight the importance of understanding the genotype‒phenotype correlations in NF1 to provide effective genetic counseling and patient management. Accordingly, revised criteria were published in 2021 that included information on genetic testing as well as clinical characteristics^11^.

The Japan Clinic Network (NF1-JNET), established by the Japanese Society of Recklinghausen Disease (JSRD), consists of 10 centers nationwide. Specialized NF1 clinics are essential for patients with severe or complex symptoms of the disease. Moreover, the importance of a coordinated hospital-based care system for patients with NF1 has become apparent^12^. An understanding of population-specific genetic characteristics is critical for genetic counseling and the development of targeted management strategies for patients with NF1.

Previous studies have demonstrated the utility of genetic testing in the molecular diagnosis of NF1 patients^13–18^. However, comprehensive studies on genotype‒phenotype characteristics in Japanese patients with NF1, especially those with malignant tumors, are limited. This study aimed to update and evaluate the genotypic and phenotypic characteristics of NF1. Our findings may improve surveillance strategies and optimize hospital-based care systems for patients with NF1.

## Materials and Methods

### Participants

Patients with clinically diagnosed or suspected NF1 were enrolled from a hereditary tumor cohort of a multi-institutional, hospital-based registry in which DNA and clinical information were collected between December 2020 and July 2024 (Mid-West Japan Hereditary Tumor Cohort, https://cgm.hsc.okayama-u.ac.jp/en/cohort/). All participants underwent genetic counseling and comprehensive *NF* genetic testing at a clinical laboratory. The inclusion criterion was meeting at least one of the seven items of the revised NIH criteria^3^.

This study was conducted in accordance with the Declaration of Helsinki, and the study protocol was approved by the Institutional Review Board of Okayama University (IRB numbers 1911-034 and 2301-026). Written informed consent was obtained from all participants.

### Sequencing and copy number analysis

Genomic DNA was extracted from peripheral blood samples, and NF panel testing via hybrid capture-based next-generation sequencing (NGS) was conducted at a clinical laboratory (Kazusa DNA Research Institute, https://www.genetest.jp/). The NF panel covered the protein-coding regions and their boundaries with introns (up to 10 bases) of five genes (*NF1*, *NF2*, *SPRED1*, *SMARCB1*, and *LZTR1*). The obtained DNA sequences were compared with publicly available human genome reference sequences (GRCh38/hg38). Variants not listed in the population database gnomAD (https://gnomad.broadinstitute.org/) and those with a frequency of less than 0.1% were also identified. Interpretation of the pathological significance of the variants was based on the American College of Medical Genetics and Genomics/Association for Medical Pathology (ACMG/AMP) variant classification guidelines^19^. Pathogenic variants of *NF1* also include copy number variants (CNVs) that cannot be detected by sequencing-based methods. For patients with negative NF panel test results, chromosomal microarray analysis (CMA) or multiplex ligation-dependent probe amplification (MLPA) was performed. CMA was performed using a test that has been included in universal health insurance coverage in Japan since 2021. MLPA was performed for *NF1* in a clinical laboratory (Ambry Genetics, https://www.ambrygen.com/). The choice between CMA and MLPA was based on sample availability and clinical indications. CMA was also performed for confirmation in cases where hybrid capture-based NGS suggested multiple exon deletions.

### Clinical data collection from the hereditary tumor cohort

NF1-associated clinical manifestations were comprehensively assessed by conducting patient interviews and reviewing medical records. The clinical data collected from each participant encompassed a wide spectrum of NF1-related features, adhering to established diagnostic criteria.

The clinical information in our cohort included cutaneous manifestations such as café-au-lait spots, axillary freckling, and cutaneous neurofibromas diagnosed as NF1. The ophthalmological evaluations focused on Lisch nodules and optic pathway gliomas. Skeletal abnormalities, such as scoliosis, limb bone deformities, and bone loss, were also recorded. NF-related benign tumors include schwannomas and plexiform neurofibromas, while malignant tumors such as malignant peripheral nerve sheath tumors, breast cancer, gastrointestinal stromal tumors, and pheochromocytomas have also been documented. The neurological and neurodevelopmental assessments included attention deficit hyperactivity disorder (ADHD), learning disabilities, autism spectrum disorder, and epilepsy. Additional clinical features, such as short stature (height >2.0 standard deviations below the mean for age and sex) and hypertension, were also recorded. Moreover, the follow-up data of each patient with NF1 and their surveillance history were documented, with a focus on MRI examinations.

## Results

### Participant characteristics

Of the 44 patients, 39 (88.6%) met the revised NIH criteria. The characteristics of the 36 patients with *NF1* germline pathogenic variants are described in Table 1. The data revealed a relatively balanced sex distribution with a slight predominance of females. There was a wide age range at the first visit, with adults ( ≥ 18 years) comprising 69.4% (25/36) of the study participants, indicating varied onset or recognition of NF1 symptoms. Family history analysis revealed that 17 of 31 unrelated individuals (54.8%) had a family history of NF1. Imaging surveillance trends showed that whole-body MRI was typically performed in adults, whereas head MRI was more common in pediatric patients.

**Table 1:** Characteristics of patients with *NF1* germline pathogenic variants.

Characteristic		Patients, No. (%)
		(*n* = 36)
Age of first visit		
mean ± sd (range), y		34.0 ± 20.3 (2–76 y)
Sex	Male	17 (47.2)
	Female	19 (52.8)
Adult ( ≥ 18 years old)		25 (69.4%)
Child ( < 18 years old)		11 (30.6%)
Family history of NF1 (*n* = 31)		
exclude related individuals	Yes	17 (54.8)
	No	14 (45.2)
Follow-up time		
median (range), days		266 (0–1057 days)
Age of MRI follow-up		
head only (median, range, %)		7 (15, 6–62, 18.9)
whole-body (median, range, %)		26 (33, 4–64, 72.2)

### Molecular characteristics

**Fig. 1:**
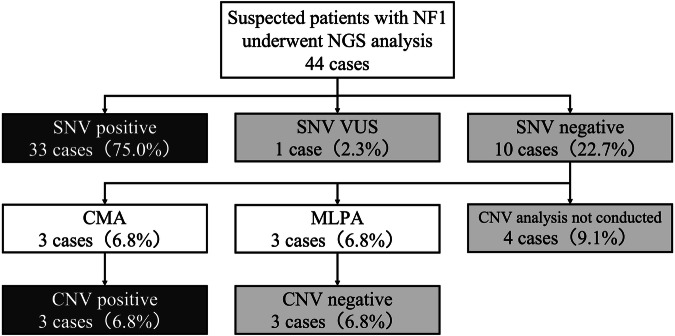
Genetic diagnosis scheme and detection rate of *NF1* pathogenic variants in patients with suspected NF1. This diagram illustrates the genetic testing process of patients with suspected NF1. A total of 44 patients who underwent NF panel testing via next-generation sequencing (NGS) were included, and 33 (75.0%) tested positive for single-nucleotide variants (SNVs). One patient had a variant of uncertain significance (VUS). The SNV-negative patients (22.7%) underwent further testing: three patients (6.8%) underwent chromosomal microarray analysis (CMA), all of whom harbored copy number variants (CNVs). Three patients (6.8%) underwent multiplex ligation-dependent probe amplification (MLPA), the results of which were negative for all three patients, and four patients (9.1%) did not undergo CNV analysis.

Genetic testing was performed on 44 patients (Fig. 1). Among these patients, 33 (75.0%) harbored germline single-nucleotide variants (SNVs), as determined by NF panel testing. One patient had a variant of uncertain significance, which was excluded from the genotypic and phenotypic analyses in this study. Among the 10 patients with negative NF panel test results, 3 (6.8%) were identified as carrying large deletions via the CGH array. The remaining three patients had negative results for MLPA. Among the patients who met the revised NIH criteria, a high germline pathogenic variant detection rate of 92.3% (36/39) was recorded in our study.

**Fig. 2:**
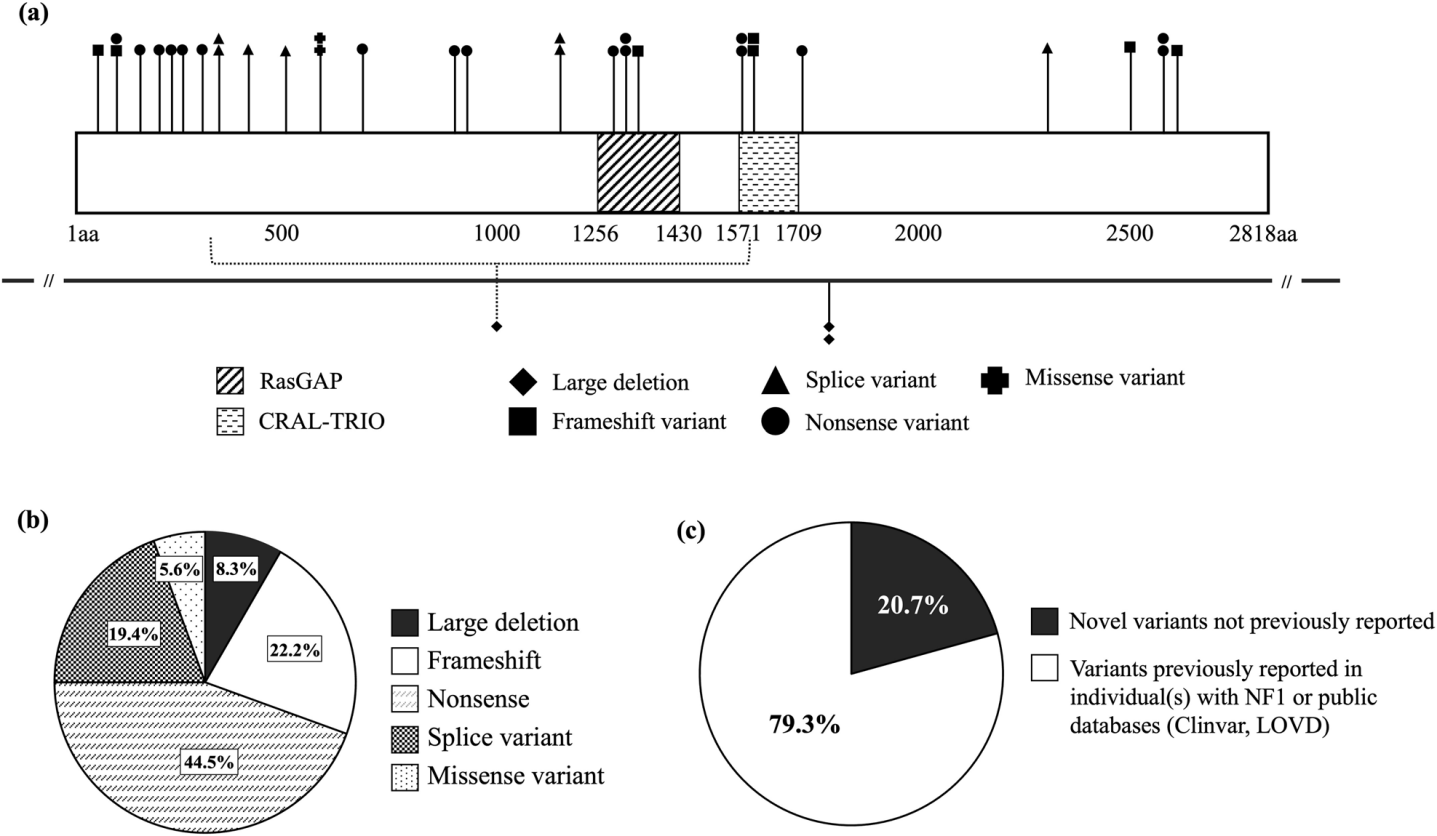
Distribution of the identified *NF1* germline pathogenic variants. **a** Schematic structure of *NF1* showing the locations of the identified variants. The RasGAP and CRAL-TRIO domains are shown in the diagonal line and dotted line boxes, respectively. The number of amino acids (aa 1–2818) is shown in the box. Amino acid numbers for each region/domain were obtained from InterPro (https://www.ebi.ac.uk/interpro/). There were no specific hotspots within *NF1*. **b** Pie chart illustrating the frequency of each variant type. Large deletions (*n* = 3, 8.3%), frameshift variants (*n* = 8, 22.2%), nonsense variants (*n* = 16, 44.5%), splice variants (*n* = 7, 19.4%), and missense variants (*n* = 2, 5.6%) are depicted. **c** Pie chart showing the novelty of the identified variants. Analysis of the variants revealed that 20.7% (6/29) were novel variants. The remaining 79.3% (23/29) were variants previously reported in individuals with NF1 or included in public databases (ClinVar, LOVD).

Analysis of variant distribution revealed no specific hotspots within *NF1* (Fig. 2a). The spectrum of variants detected included 3 deletions, 8 frameshift variants, 16 nonsense variants, 7 splice variants, and 2 missense variants (Fig. 2b). Among the 29 SNVs, 6 (20.7%) were identified as novel variants, including 4 frameshift (p.Leu104Phefs*3, p.Ser146Argfs*19, p.Asn2512Thrfs*15, p.Asp2591Valfs*13) and 2 nonsense (p.Tyr227_Pro228delins*, p.Leu972*) variants (Fig. 2c), all resulting in premature termination codons. In one family, the father (Patient 27 in Table 2) was found to carry a pathogenic variant with a variant allele frequency (VAF) of 19.9% (read count: 78/392), whereas his son (Patient 26) carried the same pathogenic variant with a VAF of 46.7% (read count: 240/514). These findings indicate that Patient 27 harbors the pathogenic variant in a mosaic form, whereas his son carries the pathogenic variant in a nonmosaic form. No pathogenic variants were identified in the *NF2*, *SPRED1*, *SMARCB1*, or *LZTR1* genes.

**Table 2:** Summary of the genotypes and phenotypes of patients with NF1.

Patient	Family			ACMG/AMP	Clinical findings									Family History	Follow-up
Num.	Num.	Variant	Protein	classification	Sex	Age of first visit	Skin	Eye	Bone	Benign tumor	Malignant tumor	Neurological	Other	(Yes/No)	days (days)
1	1	c.206_207del	p.Arg69Asnfs*7	P	F	32	CS, AF, CN	LN	—	—	—	—	—	Yes	175
2	2	c.311 T > G	p.Leu104*	P	M	76	CS, AF, CN	—	—	—	GIST	—	—	Yes	54
3	3	c.311dup	p.Leu104Phefs*3	LP	M	31	CS, AF, CN	—	—	PN	MPNST	—	—	Yes	856
4	4	c.438_439delinsA	p.Ser146Argfs*19	LP	F	13	CS	—	—	PN	—	—	—	No	786
5	5	c.574 C > T	p.Arg192*	LP	F	12	CS, AF	—	—	—	—	—	—	Yes	184
6	6	c.625 C > T	p.Gln209*	P	M	2	CS, AF, CN	—	—	Sch, PN	—	—	—	No	807
7	7	c.681_682delinsGT	p.Tyr227_Pro228delins*	LP	F	12	CS, AF, CN	—	Sco	—	—	LD	—	No	84
8	8	c.943 C > T	p.Gln315*	P	F	48	CS, AF, CN	—	Sco	PN	—	—	—	Yes	154
9	9	c.1062+1 G > A	NA	P	M	16	CS, AF	—	—	—	—	ASD	HT	Yes	287
10	-	c.1062+1 G > A	NA	P	M	46	CS, AF	—	—	—	—	—	HT	Yes	119
11	10	c.1260+5 G > C	NA	LP	M	45	CS, AF, CN	—	LBD	Sch	PCC	—	HT	Yes	406
12	11	c.1527+4_1527+7del	NA	P	F	59	CS, AF, CN	—	—	Sch	DPC	—	HT	Yes	245
13	12	c.1748 A > G	p.Lys583Arg	P	M	58	CS, AF, CN	—	—	PN	—	—	HT	Yes	147
14	-	c.1748 A > G	p.Lys583Arg	P	F	63	CS, AF, CN	—	—	—	BC	—	HT	Yes	231
15	13	c.2041 C > T	p.Arg681*	P	M	48	CS, AF, CN	—	Sco	—	LMS, BT	—	SS, HT	No	189
16	14	c.2734 C > T	p.Gln912*	P	F	11	CS	LN	Sco	PN	—	Epi	—	No	464
17	15	c.2914del	p.Leu972*	LP	F	52	CS, AF, CN	—	Sco	PN	—	—	—	Yes	1008
18	16	c.3198–2 A > G	NA	P	F	61	CS, AF, CN	—	—	Sch, PN	MPNST	—	HT	Yes	989
19	-	c.3198-2 A > G	NA	P	F	35	CS, AF, CN	—	—	Sch, PN	—	—	—	Yes	917
20	17	c.3888 T > A	p.Tyr1296*	P	F	61	CS, AF, CN	—	BL	PN	—	—	HT	No	211
21	18	c.3916 C > T	p.Arg1306*	P	F	39	CS, AF, CN	—	—	—	—	—	—	No	182
22	19	c.3916 C > T	p.Arg1306*	P	F	57	CS, AF, CN	—	—	—	BC	—	—	Yes	109
23	20	c.3947_3948del	p.His1316Argfs*2	P	M	27	CS, AF, CN	LN,OPG	BL	PN	—	—	—	Yes	294
24	21	c.4812 C > A	p.Tyr1604*	P	F	22	CS, AF, CN	LN	Sco	PN	—	—	—	Yes	995
25	-	c.4812 C > A	p.Tyr1604*	P	F	27	CS, AF, CN	—	—	—	—	—	—	Yes	217
26	22	c.4914_4917del	p.Lys1640Glyfs*36	P	M	5	CS, AF	—	—	Sch, PN	—	ADHD, LD, Epi	—	Yes	371
27	-	c.4914_4917 = /del	p.Lys1640Glyfs*36	P	M	33	CS, AF	—	—	—	—	—	—	Yes	371
28	23	c.5234 C > G	p.Ser1745*	P	F	53	CS, AF, CN	—	—	—	BC	—	—	No	91
29	24	c.6999+2 T > C	NA	P	M	9	CS, AF	LN	—	Sch, PN	—	—	—	No	784
30	25	c.7535del	p.Asn2512Thrfs*15	LP	F	18	CS, AF	LN	—	Sch	—	—	—	No	1057
31	26	c.7739 C > A	p.Ser2580*	P	M	3	CS	LN	—	—	—	LD	—	Yes	0
32	27	c.7739 C > A	p.Ser2580*	P	F	39	CS, AF, CN	—	—	Sch	—	—	—	Yes	0
33	28	c.7770_7771dup	p.Asp2591Valfs*13	LP	M	16	CS, AF, CN	—	—	PN	—	ADHD	HT	No	238
34	29	arr[GRCh37]17q11.2(29526577_29602157) x1		P	M	7	CS, AF	LN	Sco	—	—	ADHD	—	No	546
35	30	arr[GRCh37]:17q11.2(29033882_30326958) x1		P	M	27	CS, AF, CN	—	Sco	Sch	—	LD	—	No	434
36	31	arr[GRCh37]17q11.2(29033882_30367214) x1		P	M	22	CS, AF, CN	LN	S,LBD	Sch, PN	GIST	ADHD, LD, ASD	HT	No	1029

ACMG/AMP classification, *P* Pathogenic, *LP* Likely pathogenic, Sex: *F* Female, *M* Male.

Skin, *CS* Café-au-lait spots, *AF* Axillary freckling, *CN* Cutaneous neurofibroma.

Eye, *LN* Lisch nodules, *OPG* Optic pathway glioma.

Bone, *Sco* Scoliosis, *LBD* Limb bone deformity, *BL* Bone loss.

Benign tumor, *Sch* Schwannoma, *PN* Plexiform neurofibroma.

Malignant tumor, *MPNST* Malignant peripheral nerve sheath tumor, *BC* Breast cancer, *GIST* Gastrointestinal stromal tumor, *PCC* Pheochromocytoma, *DPC* duodenal papillary cancer, *LMS* Leiomyosarcoma, *BT* Bone tumor.

Neurological, *ADHD* Attention deficit hyperactivity disorder, *LD* Learning disability, *ASD* Autism Spectrum Disorder, *EPi* Epilepsy.

Other, *SS* Short stature, *HT* Hypertension.

### Genotype‒phenotype correlations in genetically diagnosed NF1 patients

The genotype‒phenotype characteristics of 36 patients genetically diagnosed with NF1 are described in Table 2. Our analysis revealed a wide spectrum of pathogenic *NF1* variants, including missense, nonsense, frameshift, and splice site variants, and deletions detected by NGS, reflecting the complex nature of *NF1* variants.

Cutaneous manifestations were prominent, with café-au-lait spots being the most common feature, present in nearly all patients, followed by axillary freckling. These findings are consistent with the established diagnostic criteria for NF1. Benign tumors, particularly schwannomas and plexiform neurofibromas, were observed in 60.0% (15/25) of the adult patients in our cohort, whereas malignant tumors were observed in 40.0% (10/25). These malignancies included female breast cancer (20.0%, 3/15), malignant peripheral nerve sheath tumors (8.0%, 2/25), gastrointestinal stromal tumors (8.0%, 2/25), and pheochromocytoma (4.0%, 1/25). One-third of patients (33.3%, 12/36) presented with skeletal abnormalities. Scoliosis was the most common skeletal issue, occurring in 9 of 36 patients, representing 25% of the entire cohort. Various neurological manifestations included learning disabilities (13.9%, 5/36), attention deficit hyperactivity disorder (11.1%, 4/36), and autism spectrum disorder (5.6%, 2/36). Cardiovascular complications, particularly hypertension, were identified in 30.6% (11/36) of the patients. Three patients with large deletions in *NF1* (Patients 34, 35, and 36) presented with a triad of cutaneous, skeletal, and neurological manifestations. The clinical outcomes of hypertension included sudden cardiac death in one patient (Patient 13) and development of a pheochromocytoma in another patient (Patient 11).

Although clear correlations between specific variants and phenotypes were not immediately apparent, patients with deletions detected by CMA appeared to exhibit more severe phenotypes, including multiple tumor types and neurodevelopmental issues.

## Discussion

Many pathogenic variants have been reported, but hotspots for *NF1* have not been identified to date^20,21^. Our cohort study revealed six novel variants. The frequency of malignancy and hypertension was greater in our cohort than that reported in previous studies, highlighting critical considerations for the surveillance of patients with NF1. These findings contribute to the growing body of knowledge on NF1 and underscore several important aspects of the disease in this population.

Pathogenic variants were detected in 92.3% (36/39) of patients meeting the revised NIH criteria for NF1, which is consistent with detection rates reported in previous studies^16,22–24^. Furthermore, we identified six novel variants, accounting for 20.7% of all variants. The frequencies of novel variants reported in previous studies have varied, which could be attributed to differences in cohort size, detection methods, and ethnic backgrounds. For example, Kiraz et al. reported 25% novel variants in their Turkish cohort via NGS analysis^25^. *NF1* is a relatively large gene comprising 60 exons, with more than 3000 different disease-causing variants described in the literature^26^. The novel pathogenic variants identified in our study indicate that NF1 variants are diverse and provide important data for genetic testing.

While our study did not reveal clear correlations between specific SNVs and phenotypes across all patients with NF1, patients with large deletions detected by CMA presented more variable phenotypes. Truncating variants can trigger nonsense-mediated mRNA decay, whereas large deletions lead to the complete loss of one gene copy. In our cohort, skeletal and neurological manifestations were associated with variants at various locations. Patients with large deletions (*n* = 3) presented a combination of cutaneous, skeletal, and neurological manifestations, which is consistent with previous reports on *NF1* microdeletion syndrome^9,26,27^. However, the small sample size of both groups limits our ability to draw definitive conclusions about these phenotypic differences. This observation supports the growing body of evidence for genotype‒phenotype correlations in NF1 and highlights the potential utility of comprehensive genetic testing methods, including CNV analysis.

We identified a mosaic variant with a VAF of 19.9% in one patient (Patient 27), whose phenotype was limited to café-au-lait spots and axillary freckling. In contrast, his child (Patient 26) carried the same variant with a VAF of 46.7% and presented with diverse manifestations, including plexiform neurofibroma and neurological symptoms, suggesting germline mosaicism in the father. This finding has important implications for genetic counseling and highlights a potential mechanism for intrafamilial phenotype variability in NF1. Germline mosaicism may explain why individuals within the same family can exhibit different severities of NF1, even when they carry the same variant.

Our data demonstrated a high prevalence of benign tumors (60.0%) and a notable occurrence of malignant tumors (40.0%), emphasizing the need for vigilant tumor surveillance in adult patients with NF1. The European Reference Network for Genetic Tumor Risk Syndromes (ERN GENTURIS) tumor surveillance guidelines recommend performing whole-body MRI at least once during the transition from childhood to adulthood^28^. Similarly, Japanese guidelines conditionally recommend whole-body MRI for patients with NF1 who have plexiform neurofibromas^29^. Previous studies have reported that the lifetime risk of malignant peripheral nerve sheath tumors (MPNSTs) in patients with NF1 is 8–15%^30–32^. In our cohort, the lower incidence of MPNSTs may be attributed to the short follow-up period (median observation period: 266 days). Our study revealed a female breast cancer incidence rate of 20.0%, which is higher than that reported previously (2.9%)^32^. This finding supports the international guideline recommendation (23, 28) for yearly MRI screening in patients aged between 30 and 50 years. Previous reports have shown that 15–20% of patients with NF1 develop arterial hypertension^33,34^, while hypertension was detected in 30.6% of our patients, with 80% of the cases occurring in adulthood. Given that one patient with hypertension experienced sudden death and another developed pheochromocytoma, regular follow-up for NF1 patients with hypertension is crucial.

While our study provides valuable insights, it is limited by its relatively small sample size and the cross-sectional nature of data collection. Some adult patients with NF1 may not have been adequately examined for ocular symptoms such as iris nodules. Longitudinal studies with larger cohorts would be beneficial to elucidate the genotype‒phenotype correlations and the natural history of NF1. Additionally, functional studies of the novel variants identified in this study could provide deeper insight into their pathogenicity and potential impact on NF1 protein function.

The strengths of our study include the assessment of the genetic landscape and clinical manifestations of NF1 in this population, which contributes to a broader understanding of this complex disorder. The high detection rate of *NF1* pathogenic variants (92.3%), including six novel variants, in patients meeting the revised NIH criteria underscores the genetic heterogeneity of NF1 and the importance of comprehensive genetic testing. Our findings also suggest potential genotype‒phenotype correlations, particularly in patients with large deletions detected via CMA. Furthermore, patients with *NF1* germline pathogenic variants exhibited a wide range of clinical features, including a high prevalence of malignant tumors and hypertension. These findings emphasize the need for hospital-based care systems and surveillance strategies in patients with NF1.
